# The Role of Methionine Sulfoxide Reductases in Oxidative Stress Tolerance and Virulence of *Staphylococcus aureus* and Other Bacteria

**DOI:** 10.3390/antiox7100128

**Published:** 2018-09-28

**Authors:** Vineet K. Singh, Kuldeep Singh, Kyle Baum

**Affiliations:** 1 Department of Microbiology and Immunology, A.T. Still University of Health Sciences, Kirksville, MO 63501, USA; krbaum@atsu.edu; 2 Mayo Clinic, Rochester, MN 53905, USA; singh.kuldeep@mayo.edu

**Keywords:** MSRA, MSRB, oxidative stress, virulence

## Abstract

Methionine sulfoxide reductases (MSRA1 and MSRB) are proteins overproduced in *Staphylococcus aureus* during exposure with cell wall-active antibiotics. Later studies identified the presence of two additional MSRA proteins (MSRA2 and MSRA3) in *S. aureus*. These MSR proteins have been characterized in many other bacteria as well. This review provides the current knowledge about the conditions and regulatory network that mimic the expression of these MSR encoding genes and their role in defense from oxidative stress and virulence.

## 1. Methionine Sulfoxide Reductases

The presence of reactive oxygen species (ROS) is potentially damaging to all cellular macromolecules. Oxidizing agents, such as hydrogen peroxide (H_2_O_2_), superoxides, and hydroxyl radicals, oxidize the sulfur atom of methionine residues, resulting in methionine sulfoxide (MetO) that typically leads to loss of protein function [1,2]. In 1981, an enzyme capable of reducing protein-bound methionine sulfoxide was identified [3,4]. These oxidized MetO residues are reduced back to methionine by methionine sulfoxide reductase (MSR) enzymes that restore normal protein functions [5,6]. Oxidation of methionine results in two stereoisomeric forms of MetO (R-MetO and S-MetO), which are reduced by two different MSR enzymes (MSRA and MSRB). MSRA is specific to S-MetO, and MSRB is specific to R-MetO [7,8,9,10]. The MetO/Met, MSRA/B-mediated oxidation and reduction of methionine residues are thus an important antioxidant mechanism [11], and methionine is no longer needed just for protein initiation [12].

## 2. MSRA and MSRB Enzymes in *Staphylococcus aureus* and Other Bacteria

In the *S. aureus* chromosome, there are three *MSRA* genes (*MSRA1*, *MSRA2* and *MSRA3*) and one *MSRB* gene. These four *MSR* genes are expressed from three different promoters. The *MSRA1* and *MSRB* genes are co-expressed as part of a polycistronic message, and the *MSRA2* and *MSRA3* genes are expressed independently from their respective promoters [13]. In bacteria, most species contain at least one copy of each of the *MSRA* and *MSRB* genes. However, similar to *S. aureus*, multiple copies of MSR encoding genes have been identified in many bacteria. *Vibrio cholera* contains two *MSRA* and three *MSRB* genes distributed in its two chromosomes. *Rhizobium meliloti* contains three *MSRA* and three *MSRB* genes with one copy of each found on a plasmid [14,15]. In addition, there is no set pattern in terms of the genetic organization of *MSR* genes across species. In some bacterial species, e.g., *Escherichia coli*, *Mycobacterium tuberculosis*, *Bordetella pertussis*, *Pseudomonas aeruginosa*, *Salmonella* Typhimurium, and *S. aureus*, *MSRA* and *MSRB* genes are two separate entities. In other bacterial species, e.g., *Bacillus subtilis* and *S. aureus*, the *MSRA* and *MSRB* genes are found adjacent to each other and are co-transcribed. Interestingly, in some other species, e.g., *Neisseria gonorrhoeae*, *Neisseria meningitidis*, and *Helicobacter pylori*, the *MSRA* and *MSRB* genes are fused and translated as separate domains within a single larger polypeptide [7,16].

## 3. Methionine and MSR Enzymes as Part of an Antioxidant System

While all amino acid residues can be oxidized, their sensitivity to oxidation is variable [17]. Methionine has a much higher propensity for oxidation than other amino acids. It is present in high concentrations on the surface of certain proteins [17] and can be oxidized without affecting the functionality of proteins. In the case of *E. coli* glutamine synthase, eight of the 16 methionine residues distant to the catalytic site could be oxidized without affecting activity [17]. Similar results were also seen in another *E. coli* protein, GroEL [18]. This protein remained fully functional even when all of its 23 methionine residues were oxidized after exposure to 15 mM H_2_O_2_ for three hours. It was only when an even higher H_2_O_2_ concentration was used, causing the oxidation of cysteines and tyrosines, that the GroEL chaperonin activity was eventually reduced [18]. The scavenging ability of methionines was further exemplified when about 40% of methionine residues in *E. coli* cells were substituted with norleucine, a carbon analog of methionine in which sulfur is replaced with a methylene group. These norleucine-substituted bacterial cells were more susceptible to killing by hypochlorite and peroxide [19]. Several proteins from the human gastric pathogen *H. pylori* were shown to interact with MSR enzymes. Many of these proteins (e.g., GroEL, catalase, and recombinase) are significantly more methionine rich than other bacterial proteins and, in all likelihood, are oxidized under oxidative stress and salvaged by the activity of MSR enzymes [20]. Thus, MSR proteins not only repair oxidative damage to methionine residues through the oxidation/reduction cycle but also serve as scavengers of ROS and protect cells from more widespread oxidative damage [11,17].

## 4. Environmental Impact on *MSR* Expression 

In *S. aureus*, MSRA1 and MSRB proteins were first identified as proteins overproduced in response to the presence of cell wall-active antibiotics [21]. Later, the expression of *MSRA1* and *MSRB* genes were determined to be induced at the transcriptional level and specifically in response to cell wall-active antibiotics [22,23]. Using a promoter-*lacZ* fusion, it was shown that none of the *S. aureus MSR* genes were induced under conditions of oxidative stress [24]. Cell wall-active antibiotics only induced the expression of the *MSRA1*/*MSRB* locus and had no effect on the expression of *MSRA2* or *MSRA3* genes [24]. A high salt concentration caused some induction of *MSRA3* but not of the other *S. aureus MSR* genes [24]. The expression of *MSRA2* and *MSRA3* genes was very low, and both were expressed higher during the early exponential phase of bacterial growth [24]. The expression of the *MSRA1*/*MSRB* locus occurs at a much higher level than the expression of *MSRA2* and *MSRA3* and is most expressed during the late log and stationary phases of growth [24]. Surprisingly, cell wall-active antibiotics induce the expression of *MSRA1* and *MSRB* genes in *S. aureus*, but these MSR enzymes appear to play no direct role in defense from these antibiotics [13]. It is speculated that cell wall-active antibiotics destabilize the cell wall and allow the oxidants easy access to cellular macromolecules. MSRA1 and MSRB might be needed at higher concentrations during exposure of *S. aureus* to the cell wall inhibitors.

When the role of SigmaB (stress responsive sigma factor) was investigated, it enhanced, but was not required for, the expression of the *S. aureus MSRA1*/*MSRB* locus [25,26]. In addition, induction of the *MSRA1*/*MSRB* locus by cell wall-active antibiotics was inhibited by glycerol monolaurate that interfered with signal transduction pathways. This result led to the speculation that an unidentified signal transduction pathway might be involved in the induced expression of *MSRA1*/*MSRB* in the presence of cell wall-active antibiotics [26]. More recently, a higher level of expression of *MSRA2* gene was observed in *S. aureus* after sunlight exposure in both oxic and anoxic conditions. Thus, it has been speculated that MSRA probably defends the bacteria from oxygen-dependent and oxygen-independent photostresses [27].

Expression of *MSR* genes in other pathogenic species has also been investigated. Similar to *S. aureus MSRA1*/*MSRB*, in *H. pylori* [20], *E. coli* [28], and *B. subtilis* [29] the late log and stationary phase cultures of these bacteria had the highest MSR activity. In addition to *MSRA1*/*MSRB* induction by cell wall-active antibiotics, piplartine, a biologically active alkaloid from peppers [30], showed enhanced *MSR* expression in the pathogenic parasite, *Trypanosoma cruzi* [31].

There is no doubt that MSRs protect cells from oxidative stress, but there are only a handful of species where oxidative stress conditions have been shown to induce the expression of *MSR* genes. In *Streptococcus gordonii*, H_2_O_2_ induced expression of the *MSRA* gene [32]. Oxidative stress conditions induced the expression of *MSRA* in the xenobiotics-degrading bacterium *Ochrobactrum anthropic* [33]. The expression of *MSRA* gene in plant bacterium *Xanthomonas campestris pv. phaseoli* was highly induced by exposure to oxidants and N-ethylmaleimide [34]. In *P. aeruginosa*, H_2_O_2_, paraquat, or sodium hypochlorite had no effect on *MSRA1* expression, but expression of *MSRB* was induced by sodium hypochlorite [35]. Oxidative stress conditions caused transcriptional upregulation of *MSR* in *H. pylori* [36]. In addition to oxidative stress, a slight upshift in pH (from 6.2 to 7.3) also led to increased expression of *MSRA* in *S. gordonii* [37]. Finally, the *B. subtilis MSRA/MSRB* operon was induced by paraquat but not by H_2_O_2_ [38].

## 5. Cellular Control of *MSR* Expression

In *S. aureus* and *B. subtilis*, the expression of *MSRA* and *MSRB* genes appears to be under the control of SigmaA [26,38]. *In B. subtilis*, a transcriptional regulator, Spx, has a positive impact on the expression of *MSRA*/*MSRB* genes [38]. Further, paraquat induces expression of *MSRA*/*MSRB* in *B. subtilis*, and this phenomenon is seen even in Spx-deficient cells, suggesting that some unidentified factors also play a role [38]. YjbH acts as a post-transcriptional negative regulator of global oxidative/thiol stress in *B. subtilis* [39], binding and stabilizing Spx [40]; thus, YjbH may be indirectly involved in the regulation of *MSRA*/*MSRB* expression in this bacterium. Spx is believed to be required for the upregulation of *MSRA1/MSRB* in *S. aureus* as well [41]. In *E. coli*, although iron has no influence on the expression of *MSRA*, an iron-responsive small RNA RyhB downregulates the expression of *MSRB* and also interferes with the translation of *MSRB* transcripts [42]. The *csrA* gene of *Enterococcus faecalis* encodes a protein with homology to MSRA and is upregulated in the presence of cadmium, mercury, lead, copper and manganese; this outcome can be used as a biosensor for monitoring heavy metals in the environment [43]. In *N. meningitidis*, *MSRA* is part of the SigmaE regulon [44], and in the rhizobacterium *Azospirillum brasilense* expression of *MSRA* is controlled by heat-shock sigma factor RpoH2 [45]. In *S. gordonii*, an amylase-binding protein A (AbpA) positively upregulates the expression of peptide MSR [46].

## 6. Defense from Oxidants

There are numerous reports that provide evidence that lack of MSR activity and, as a result, increased methionine oxidation can lead to severe growth defects and even cell death. Most studies suggest that the MSRA enzyme is critical for defense of bacterial cells from oxidative stress and that MSRB plays little-to-no role under these stresses. In *E. coli*, the catalytic activity of MSRA enzyme was 1000-fold higher than that of MSRB in reducing free methionine sulfoxide [8]. However, in *S. aureus*, MSRB was shown to have 28-fold higher activity than MSRA [22]. Nonetheless, lack of MSRB had no effect on the oxidative stress tolerance of this bacterium, and MSRA1 appeared to be more significant for defending against these conditions [13]. In addition, individual deletions of *MSRA2* or *MSRA3* genes resulted in no change in oxidative stress resistance of *S. aureus* when compared with the wild-type bacterium [13]. However, the sensitivity to oxidative stress was confounded when *S. aureus* lacked all three MSRA proteins [13]. Incubation with hypochlorous acid (HOCl) led to the oxidation of a significant fraction of methionine residues in *S. aureus* and *E. coli*. When the percentage of oxidized methionine residues increased from exposure to 150 µM HOCl, the cellular MetO content increased from 6% (untreated cells) to 22%, resulting in a 55% decrease in viability [47]. Exposure to 200 µM HOCl oxidized 40% of the cellular methionine residues and resulted in an almost complete loss of bacterial viability [47].

The significance of MSR enzymes is also apparent from the growth impairment and survival of *MSR* mutants in *E. coli* [48] and *S. gordonii* [32,37] after H_2_O_2_ treatment. In *Xanthomonas campestris pv. phaseoli*, *MSRA* mutant cells in stationary growth phase are 10–100-fold more sensitive to H_2_O_2_, tert-butyl hydroperoxide, menadione, and N-ethylmaleimide [34]. In the microaerophilic pathogen *Campylobacter jejuni*, the double *MSRA*-*MSRB* mutant showed a growth defect under *in vitro* microaerobic conditions, but the individual *MSRA* or *MSRB* mutants did not [49]. However, during conditions of oxidative and nitrosative stress, the double *MSRA*-*MSRB* mutant was markedly more sensitive, and the individual *MSRA* and *MSRB* mutants also showed increased sensitivity [49]. In *M. tuberculosis*, lack of MSRA or MSRB showed no change in resistance to oxidants, but a double mutant was vulnerable to reactive oxygen and reactive nitrogen intermediates [50]. An *MSR* mutant of *H. pylori* with no detectable MSR activity showed severe growth defects and survival in the presence of oxidants. A derivative strain of *H. pylori* that still produced MSRA but not MSRB activity remained sensitive to oxidants [36]. Inactivation of the *MSRA* gene of *S. gordonii* led to increased bacterial sensitivity to H_2_O_2_ [37]. In *P. aeruginosa*, mutations in *MSRA* and *MSRB* increased sensitivity to oxidative stress, and an *MSRA*-*MSRB* double mutant showed an additive effect in terms of oxidant sensitivity [35]. *E. faecalis* hasone MSRA and one MSRB encoding genes in different parts of the chromosome. The individual *MSRA* and *MSRB* mutants of this bacterium were sensitive to H_2_O_2_, and as in *P. aeruginosa*, mutational effects were additive in the double *MSRA*-*MSRB* mutant [51].

Oxidation of methionine residues of acid-soluble spore proteins, (SASP), in MSRA-deficient cells of *B. subtilis* reduces their ability to bind DNA, which is significant for the long-term survival of spores [52]. MSRA-deficient *S.* Typhimurium showed normal growth in broth but was sensitive to HOCl [53]. Lack of MSRAB2 in *Streptococcus pneumoniae* enhanced its killing by H_2_O_2_ [54]. While the *MSRA* mutants of *Mycobacterium smegmatis* were sensitive to the presence of oxidants, the *MSRB* mutants showed no such defect. Thus, as in *S. aureus*, MSRA and not MSRB may be the important MSR in *M. smegmatis* [55]. Even among fungal species, such as *Aspergillus nidulans*, the MSRs are important for defense against oxidants [56]. However, sometimes the protective role of MSR enzymes from oxidative stress is not as evident. In the case of the oral pathogen *Aggregatibacter actinomycetemcomitans*, no difference in growth was apparent between the wild-type and *MSR* mutant strains when exposed to H_2_O_2_ or paraquat.

## 7. Methionine Sulfoxide Reductases and Virulence

Many studies have provided evidence about the role of MSR proteins in the virulence of bacterial pathogens. Based on analysis of nucleotide sequences and the presence of synonymous vs. non-synonymous single nucleotide polymorphism, the role of MSRA enzyme has been postulated in the transition of the bacterium *Staphylococcus epidermidis* from commensalism to pathogenicity [57]. In *S. aureus*, recent findings suggest that MSRA1 plays a role in virulence, but other MSRs (MSRA2, MSRA3, and MSRB) play almost no such role. Strains of *S. aureus* deficient in MSRA1 showed reduced hemolysis and were less adherent to human lung epithelial cells [13]. These MSRA1-deficient cells also showed a decrease in survival in mouse tissue when compared with wild-type *S. aureus* [13]. Further, reduced MSRA activity in many bacteria impacted survival inside phagocytic cells [47,53,58,59].

MSRA was shown to be required for the full virulence of the plant pathogen *Erwinia chrysanthemi* [60]. The presence of MetO in the gut increased the viability of *Drosophila* infected with *V. cholera* [61]. MetO in enterocytes probably competes with protein-bound MetO, titrating away the host MSRs that favors the *Drosophila* and this in turn reduces the virulence of *V. cholera* [61]. In *S.* Typhimurium, MSRA but not MSRB has an important role in oxidative stress protection and virulence [62]. Deletion of *MSRA* in *S.* Typhimurium led to very little colonization of poultry [63]. Further, in *S.* Typhimurium, MSRB has only a weak activity against peptidyl R-MetO, but MSRA shows activity against both free and peptidyl S-MetO [62]. In this bacterium, an additional gene, *MSRC*, is present that codes for a protein that reduces only free R-MetO and has been shown to be essential for survival in the presence of H_2_O_2_ inside macrophages and in mice [62].

In *E. faecalis*, individual *MSRA* and *MSRB* mutants were also sensitive to activated macrophages and were attenuated in the *Galleria mellonella* insect infection assay [51]. In another study, MSRB was shown to have a role in the virulence of *E. faecalis* [51]. Mutations in *MSRA* and *MSRB* genes in *P. aeruginosa* decreased virulence in a *Drosophila melanogaster* infection model, and a double *MSRA-MSRB* mutant showed an additive effect for reduction in virulence [35]. An *MSRA* mutant of *M. smegmatis* had more reduced survival inside mice macrophages than the wild-type strain [64]. However, an *MSRB* mutant of *M. smegmatis* showed only a moderate reduction in survival inside the macrophages. This supports again that MSRA, not MSRB, is the important MSR in *M. smegmatis* [55]. MSR-deficient *H. pylori* showed severe reduction in the colonization of mouse stomach [36], and loss of MSRAB2 in *S. pneumoniae* dramatically reduced virulence and enhanced its uptake by macrophages [54].

Factors contributing to decreased virulence may arise from reduced colonization efficiency of MSR-deficient bacteria. Further, lack of MSR enzymes may compromise the integrity of the bacterial surface proteins responsible for adherence to eukaryotic cells [15,16,54,65]. MSRA in *Mycoplasma genitalium* was shown to affect adherence to sheep erythrocytes, and in the absence of this enzyme, the bacterium could not grow in hamsters [65]. Lack of MSRA also reduced cytotoxicity of *M. genitalium* to cervical epithelial cells and monocytic cells [66]. In terms of cellular localization, the *S. gordonii* MSRA was detectable in the cell wall fraction and probably played a role in repair of proteins, such as SspAB adhesins [32]. The *MSRA* mutant of *S. gordonii* was significantly impaired in fibronectin binding, which is significant for initial colonization of human tissues [67].

The *MSRA* and *MSRB* genes in *Francisella tularensis* are located in different regions of the chromosome. Surprisingly, MSRB not MSRA appears to be a key determinant of virulence in *F. tularensis*. Lack of MSRB reduces growth and resistance to oxidants and macrophages, but lack of MSRA produces no such effect. However, the lack of MSRA reduces the in vivo number of *F. tularensis* in mouse tissues, but the effect is much more pronounced in the absence of MSRB [68]. In an in vivo competition experiment, the *MSRB* mutants of *Lactobacillus reuteri* showed an ecological fitness and were recovered in smaller numbers than their wild-type counterparts in the gut of 50% of the inoculated mice [69]. Even in the parasite *Leishmania major*, a mutation in the *MSRA* gene increased sensitivity to H_2_O_2_ and decreased infectivity in macrophages [70]. However, these mutants induced normal lesions in BALB/c mice, suggesting a limited or no role in virulence [70].

Some reports, however, have questioned the role of MSR proteins in bacterial virulence. MSRA was initially thought to play a role in adherence of *N. gonorrhoeae* to eukaryotic cells [16]. In *N. gonorrhoeae*, two forms of MSRA/B are produced: one has a signal peptide that gets localized to the outer membrane and the other lacks the signal peptide but remains in the cytoplasm. Even though MSRA/B conferred protection from oxidative damage, lack of MSRA/B had no effect on bacterial adherence to conjunctival cells [71]. In addition, the *MSRA* mutant of *N. gonorrhoeae* showed no reduction in sensitivity to in vitro killing by polymorphonuclear cells from BALB/c mice, and there was reduced recovery of these mutants when compared with wild-type bacteria from BALB/c infected mice after only eight days of infection [72]. A mutation in the *MSRA* gene of the oral pathogen *A. actinomycetemcomitans* abolished MSR activity, but the mutant showed no reduction in resistance to oxidative stress or adherence to epithelial cells, suggesting the MSRA system has no role for virulence in this bacterium [73]. Similar to the no change in oxidative stress tolerance in *MSRA* mutants of *A. actinomycetemcomitans*, these mutants exhibited unaltered survival inside the macrophages and adherence to the epithelial cells. These findings also suggest that MSRA may not be a virulence contributor in this pathogen [73].

## 8. Conclusions

The MSR enzymes have gained significance as key determinants of protection of bacterial species from oxidative stress and of contributors of virulence. While most literature suggests that the MSRA protein is key to these functions, little is known about the precise function of MSRB. These enzymes are overproduced in response to cell wall-active antibiotics and are considered to be important members of cell wall stress stimulon in an important bacterium, *S. aureus*. More studies are needed to understand why many bacteria need so many genes that encode these enzymes, how they are regulated, why *S. aureus* turns on the expression of these genes under cell wall-active antibiotic stress, and what their precise function is in bacterial physiology and virulence.
